# High Concentrations of H_2_O_2_ Make Aerobic Glycolysis Energetically More Favorable for Cellular Respiration

**DOI:** 10.3389/fphys.2016.00362

**Published:** 2016-08-23

**Authors:** Hamid R. Molavian, Mohammad Kohandel, Sivabal Sivaloganathan

**Affiliations:** Department of Applied Mathematics, University of WaterlooWaterloo, ON, Canada

**Keywords:** cancer cell metabolism, warburg effect, glycolysis, oxidative phosphorylation, pentose phosphate pathway, reactive oxygen species

## Abstract

Since the original observation of the Warburg Effect in cancer cells, over 8 decades ago, the major question of why aerobic glycolysis is favored over oxidative phosphorylation has remained unresolved. An understanding of this phenomenon may well be the key to the development of more effective cancer therapies. In this paper, we use a semi-empirical method to throw light on this puzzle. We show that aerobic glycolysis is in fact energetically more favorable than oxidative phosphorylation for concentrations of peroxide (H_2_O_2_) above some critical threshold value. The fundamental reason for this is the activation and high engagement of the pentose phosphate pathway (PPP) in response to the production of reactive oxygen species (ROS) H_2_O_2_ by mitochondria and the high concentration of H_2_O_2_ (produced by mitochondria and other sources). This makes oxidative phosphorylation an inefficient source of energy since it leads (despite high levels of ATP production) to a concomitant high energy consumption in order to respond to the hazardous waste products resulting from cellular processes associated with this metabolic pathway. We also demonstrate that the high concentration of H_2_O_2_ results in an increased glucose consumption, and also increases the lactate production in the case of glycolysis.

## Introduction

Increased aerobic glycolysis (the Warburg Effect) in proliferating cancer cells has been a perplexing puzzle that has remained unresolved for more than 80 years (Warburg, 1930, 1956; Gatenby and Gillies, 2004; Vander Heiden et al., 2009; Cairns et al., 2011; Schulze and Harris, 2012). The observation that cancerous cells are dominated by aerobic glycolysis is confounded by the fact that this metabolism produces far less energy compared to oxidative phosphorylation—generating 2 ATP from one molecule of glucose when compared to oxidative phosphorylation which generates 36 ATP (in the ideal case; Warburg, 1930, 1956). Initially, it was conjectured that defects in mitochondria might be the main reason for the increased aerobic glycolysis (Gatenby and Gillies, 2004; Vander Heiden et al., 2009; Cairns et al., 2011; Schulze and Harris, 2012), but successive experimental investigations have failed to confirm this scenario (Warburg, 1930, 1956).

Mitochondria produce reactive oxygen species (ROS) H_2_O_2_ in non-cancerous and cancerous cells during oxidative phosphorylation (Turrens, 2003). Moreover, in cancerous cells the concentration of H_2_O_2_ is also enhanced by the production of H_2_O_2_ through tumor suppressor and oncogenic agents (Szatrowski and Nathan, 1991; Vafa et al., 2002; Turrens, 2003; Sablina et al., 2005; Nogueira et al., 2008; Bensaad et al., 2009). The accumulation of H_2_O_2_ results in a toxic environment for cell compartments; moreover, mitochondria, as a source of H_2_O_2_, are much more vulnerable to H_2_O_2_ and as a result the development of conducible intracellular conditions can trigger tumor necrosis factors (TNF; Comporti, 1987; Schulze-Osthoff et al., 1993). As a defense mechanism, mitochondria import reduced glutathione (GSH), which is produced mainly through the activation of the pentose phosphate pathway (PPP) in the cytoplasm to detoxify the H_2_O_2_ (Deneke and Fanburg, 1989; Fernandez-Checa et al., 1997; Anastasiou et al., 2011). The removal of ROS is critical for cell survival since under high concentrations of H_2_O_2_, cell metabolism pathways are shut down in order to drive the flow of glucose to the PPP and thus produce enough GSH to detoxify the H_2_O_2_ (Anastasiou et al., 2011). However, the activation and maintenance of the PPP requires ATP hence an active, ramped up, productive cell metabolism is needed in order to produce more ATP when PPP is highly activated.

To understand the mechanism behind aerobic glycolysis and the role of ROS, we consider the following major metabolisms and detoxification pathway: oxidative phosphorylation, glycolysis, and the PPP. We assume that ATP, GSH, and H_2_O_2_ are the major players in the cell metabolism dynamics and the three chemical reactions involved are therefore (Supplementary Information),

(1) $$\begin{array}{cc} \begin{array}{l} Glucose\;+\;6O_{2}\;\to \;6CO_{2}\\ \;+\;6H_{2}O\;(\text{energy }=\;36\;ATP)\;Respiration \end{array} & \;(1) \end{array}$$

(2) $$\begin{array}{cc} Glucose\;\to \;2Lactate^{-}\;+\;2H^{+}\;(\text{energy }=\;2\;ATP)\;Glycolysis & \;(2) \end{array}$$

(3) $$\begin{array}{cc} \begin{array}{l} Glucose\;+\;ATP\;+\;H_{2}O\;+2\;GSSG\;\to \;R5P\;+\;4GSH\;+\;CO_{2}\\ \;+\;ADP\;Detox \end{array} & \;(3) \end{array}$$

In reaction (3), *R*5*P* may be used for synthesis of nucleotides and nucleic acids, which are necessary for cell proliferation, and GSH is used in the following equation to detoxify H_2_O_2_

(4) $$\begin{array}{cc} H_{2}O_{2}\;+\;2GSH\;\overset{GPx}{\to }\;GSSG\;+\;2H_{2}O & \;(4) \end{array}$$

This reaction involves intermediate steps in which GPxr interacts directly with H_2_O_2_ and GSH is a co-factor which produces GPxr (Supplementary Information). The concentration of GSH and GPxr in cells are respectively about 0.1–7 mM (Deneke and Fanburg, 1989; Li et al., 2000; Ng et al., 2007; Anastasiou et al., 2011) and 10 nM–5 μM (Antunes and Cadenas, 2001; Stone, 2004), hence it seems that there is always enough GSH to detoxify H_2_O_2_. However, at high concentration levels of H_2_O_2_, H_2_O_2_ primarily modulates the concentration levels of GSH. Cells respond to these elevated levels by producing more GSH to detoxify the accumulated H_2_O_2_ (Bellomo et al., 1992). Therefore, at high concentrations of H_2_O_2,_ the production rate of GSH depends in turn on the concentration level of H_2_O_2_ (Li et al., 2000; Ng et al., 2007). A good indication of this behavior is the full diversion of glucose flux into the PPP when cells are contaminated with high concentrations of H_2_O_2_ (Fernandez-Checa et al., 1997). Increasing the concentration of H_2_O_2_ further, eventually results in cell death. Thus, we can define an upper limit for the concentration of H_2_O_2_ above which cells go through apoptosis and we call this the cell sensitivity concentration (CSC) level. For concentrations of H_2_O_2_ much lower than CSC the production rate of GSH is very low. As the concentration of H_2_O_2_ becomes comparable to CSC, cells start to activate the PPP to produce more GSH. The produced H_2_O_2_ by cell mitochondria is a major player in this response, since they are at the center of H_2_O_2_ production and they, if functional, can activate TNF. At high concentrations of H_2_O_2_, the H_2_O_2_ produced by mitochondria does not diffuse into the cell cytoplasm and accumulates instead around the mitochondria which, as a result, activate TNF.

We include these observations in the form $P_{GSH}=\beta P_{ROS}^{mt}\;+\;\gamma P_{ROS}^{ext}$ where *P*_*GSH*_, $P_{ROS}^{mt}$, and $P_{ROS}^{ext}$ are respectively the production rates of GSH, H_2_O_2_ by mitochondria and H_2_O_2_ by external sources, and β and γ are functions of the concentration of GSH and, the difference between CSC (*C*_0_) and the concentration of H_2_O_2_ (*C*_*ROS*_). We choose β and γ to be different since, in general, the response of mitochondria to the accumulation of H_2_O_2_ differs from that of the rest of the cell. β and γ are very small for low concentrations of H_2_O_2_ and start to increase as the concentration of H_2_O_2_ increases. Close to *C*_0_ they become very large in order to drive most of the consumed glucose to the PPP pathway. Since mitochondria can activate TNF in their oxidative phosphorylation state (Schulze-Osthoff et al., 1993), there is a stronger response at high concentrations of H_2_O_2_, hence β should be significantly larger than γ.

We assume that both oxidative phosphorylation and glycolysis are activated to produce energy for cell needs and for the PPP and investigate the production rate of ATP in the presence of H_2_O_2_. The net production of ATP is the sum of the production of ATP by oxidative phosphorylation and glycolysis minus the consumption of ATP by the PPP (which primarily detoxifies the generated H_2_O_2_ by mitochondria and other sources). We obtain the production and consumption as functions of the oxygen and glucose consumption and GSH production ($P_{GSH}=\beta P_{ROS}^{mt}\;+\;\gamma P_{ROS}^{ext}$). Using $P_{ROS}^{mt}=\alpha q_{O}$, where α is the fraction of oxygen consumption (*q*_*O*_) converted to H_2_O_2_ by mitochondria [about 1/100−2/100 Turrens, 2003], thus we have (Supplementary Information):

(5) $$\begin{array}{cc} P_{ATP}\;=\;\frac{\left(\frac{17}{3}\;-\;\frac{3}{4}\;\alpha \beta \right)(q_{G}\;-\;\frac{\gamma }{4}P_{ROS}^{ext})}{1\;+\;\frac{r\alpha \beta }{4}}r\;+\;2\;q_{G}\;-\;\frac{3\gamma P_{ROS}^{ext}}{4} & \;(5) \end{array}$$

where *q*_*G*_ is the total consumption of glucose and *r* is the ratio between oxygen and metabolic glucose consumptions. We now consider the case when the net production rate of ATP exactly balances the energy requirements of the cell and derive the following result for the consumption of glucose in terms of the concentration and production rate of H_2_O_2_ (Supplementary Information):

(6) $$\begin{array}{cc} q_{G}^{SS}\;=\;\frac{(9+17r)\gamma }{24+68r\;-\;3r\alpha \beta }P_{ROS}^{ext}\;+\;\frac{12+3r\alpha \beta }{24+68r\;-\;3r\alpha \beta }q_{ATP}^{cell} & \;(6) \end{array}$$

where $q_{ATP}^{cell}$ and $q_{G}^{SS}$ are respectively the ATP and glucose consumption by the cell in the equilibrium state. Also, the total amount of lactate production for the case of pure glycolysis (*r* = 0) reads:

$$P_{Lact}^{r\;=\;0}\;=\;q_{ATP}^{cell}\;+\;\frac{\gamma }{4}P_{ROS}^{ext}$$

We first consider the special case of zero external production and low concentrations of H_2_O_2_ (αβ is very small). In this case, and for a purely glycolytic metabolism (*r* = 0), *P*_*ATP*_ = 2*q*_*G*_ which implies that 2ATP are produced for each molecule of glucose—this is the well-known case of pure glycolysis. In the case of dominant respiration (*r* = 6) *P*_*ATP*_ = 36*q*_*G*_, which is again the net energy production rate for a cell under oxidative phosphorylation. A simple comparison of 2–36 ATP production leads to the superficial, albeit prominent conclusion that, for the case of low H_2_O_2_ concentrations, oxidative phosphorylation is the most efficient metabolism.

**Figure 1:**
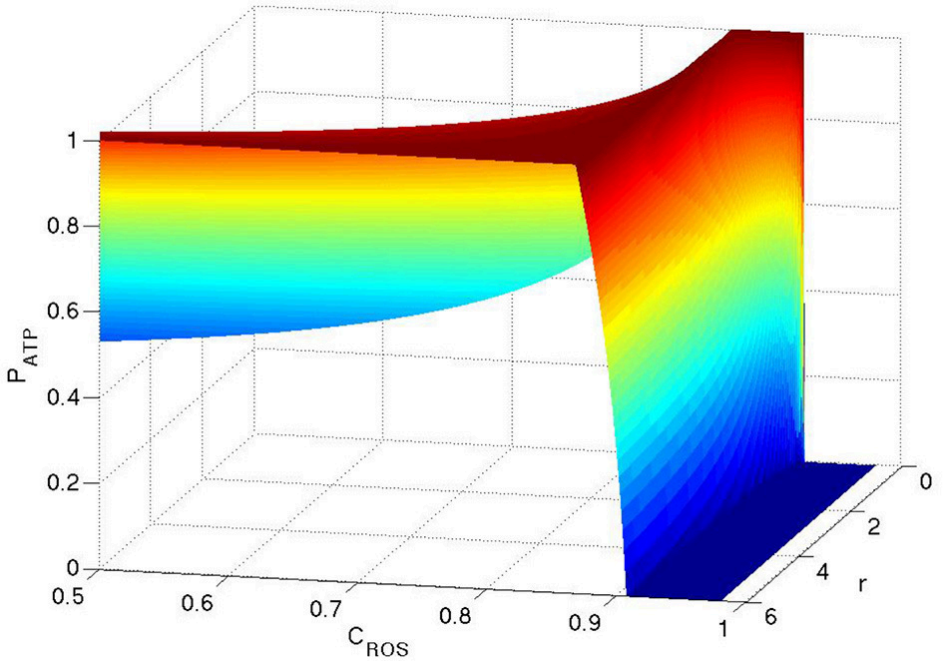
**ATP production as functions of ***C***_***ROS***_ and ***r*****. The production of ATP is normalized to one for any given concentration of ROS. Here $P_{ROS}^{ext}=\frac{q_{G}}{50}$.

We now search for the most efficient metabolism in the presence of H_2_O_2_ by finding the maximum production rate of ATP (*P*_*ATP*_) as a function of *r*. For αβ < α_*T*_, where α_*T*_ = 68/9 the maximum production of ATP arises at *r* = 6. This is in agreement with the well-known fact that oxidative phosphorylation is the most efficient way for ATP production. However, rather interestingly, for αβ > α_*T*_ the maximum production rate of ATP occurs at *r* = 0 and the transition point is independent of $P_{ROS}^{ext}$. This implies that for some concentrations of H_2_O_2_, glycolysis is in fact energetically more efficient than oxidative phosphorylation, which directly contradicts current prevailing explanations. To illustrate this metabolic transition in terms of the concentration of H_2_O_2_ we choose two functions $\beta =\frac{100}{1-C_{ROS}/C_{0}}$ and $\gamma =\frac{10}{1-C_{ROS}/C_{0}}$ and substitute these into Equation (5). These functions mimic the real behavior of the system in which the production rate of GSH increases as the concentration of H_2_O_2_ increases. The coefficient β is chosen to be larger because (a) TNF is activated by mitochondria and (b) at high concentrations of H_2_O_2_, the produced H_2_O_2_ cannot diffuse into the cell and accumulates in the vicinity of the mitochondria which occupy a much smaller space within the cell. In Figure 1 we plot the normalized *P*_*ATP*_ (*P*_*ATP*_ is normalized to be one for any given concentration of ROS) as a function of *C*_*ROS*_ and *r*. For *C*_*ROS*_ < 0.87*C*_0_, which corresponds to β < α_*T*_, the maximum production rate arises at *r* = 6 and for *C*_*ROS*_ > 0.87*C*_0_, which corresponds to αβ > α_*T*_, it transitions to pure glycolysis (*r* = 0).

As a first step to better understand the nature of the transition from oxidative phosphorylation to glycolysis, we first take note that the inefficiency observed in oxidative phosphorylation is fundamentally due to the fact that for some concentrations of H_2_O_2_, a cell must expend most of its produced ATP in detoxifying its self-generated H_2_O_2_ (by mitochondria). This is supported by the fact that for α = 0 (i.e., no production of H_2_O_2_ by mitochondria), it is respiration that is the more efficient metabolism for any concentration of H_2_O_2_. In contrast, for α≠0, no matter how small α is, the shift in metabolism (from respiration to glycolysis) occurs when concentrations of H_2_O_2_ exceed some critical threshold value. At these concentration levels of H_2_O_2_, the net produced energy by oxidative phosphorylation for one molecule of glucose is less than that produced through glycolysis. To gain a more quantitative understanding of this phenomenon, we first observe that α is a property of mitochondrial efficiency. It can safely be assumed that this remains constant independent of H_2_O_2_. Meanwhile β changes through either an increase in the concentration levels of H_2_O_2_ or a decrease in the concentration levels of GSH and thus results in a crossing of the transition point to glycolysis. In non-cancerous cells and under normal conditions, H_2_O_2_ is mainly produced by mitochondria and diffuses through the cell. In this case, the low production of GSH and other antioxidants are sufficient to detoxify the H_2_O_2_, hence normal cells function in the αβ < α_*T*_ regime. In this case, oxidative phosphorylation is the most efficient mechanism for ATP production. However, in proliferating cancer cells, H_2_O_2_ is produced by growth factors and mitochondria that work at higher rates to compensate for the increased energy needs of proliferating cells. Hence, the concentrations of H_2_O_2_ are much higher and this can push the cell into the regime αβ > α_*T*_ in which glycolysis is the more efficient metabolism.

**Figure 2:**
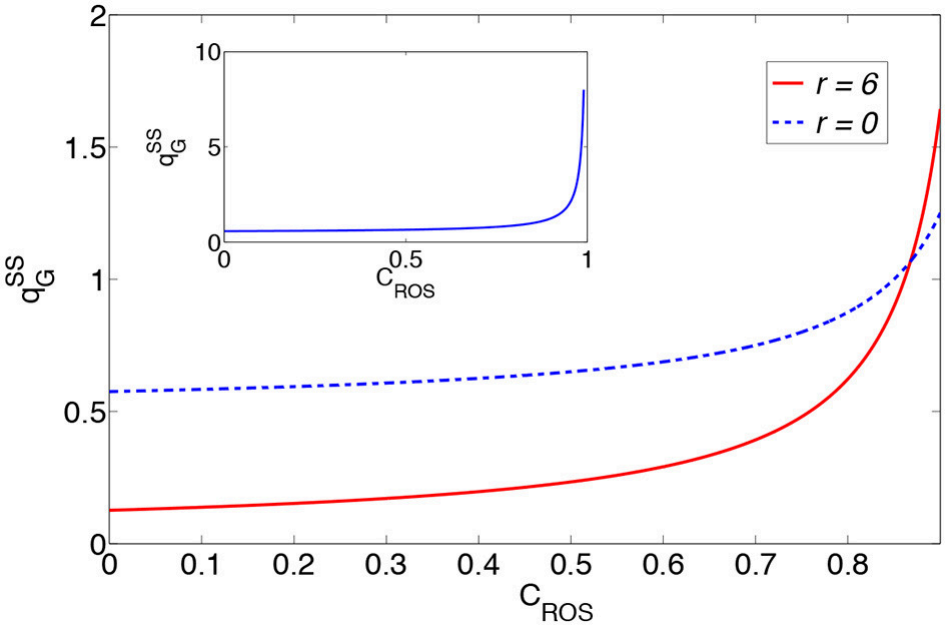
**Glucose consumption as a function of the concentration of H_**2**_O_**2**_ for ***r*** = 0 and ***r*** = 6**. Here, $P_{ROS}^{ext}=\frac{q_{G}}{50}$, $\alpha =\frac{1}{100}$, and $q_{G}^{SS}$ is in the dimension of $q_{ATP}^{cell}$. The inset shows the glucose consumption as functions of *C*_*ROS*_ for *r* = 0.

In Figure 2 we plot the glucose consumption as functions of *C*_*ROS*_ for the two cases *r* = 0 and *r* = 6 under constant cell needs. These plots show the significant increase in glucose consumption as functions of the concentration of H_2_O_2_. Therefore, the generated H_2_O_2_ by growth factors and other sources is one of the major reasons for the increase in glucose consumption. Also, the two plots intersect for *C*_*ROS*_ = 0.87*C*_0_, which means that for a given cell glucose requirement, the energy expended for the consumption of glucose through oxidative phosphorylation exceeds the corresponding expenditure when the cell has shifted to a glycolysis metabolism and this is due to the production of H_2_O_2_ by mitochondria.

**Figure 3:**
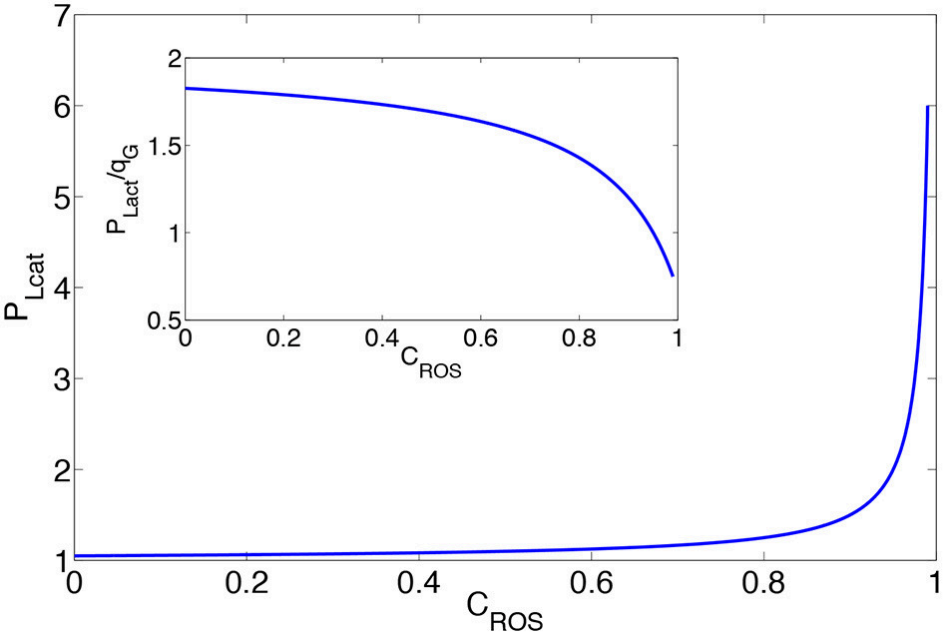
**Production of lactate as a function of the concentration of H_**2**_O_**2**_**. The inset shows the ratio between lactate production and glucose consumption. *P*_*Lact*_ is in the dimension of $q_{ATP}^{cell}$, and $P_{ROS}^{ext}=\frac{q_{G}}{50}$. In the inset, the ratio between lactate production to glucose consumption is plotted.

In Figure 3 we plot the lactate production for *r* = 0 as a function of *C*_*ROS*_ assuming that $q_{ATP}^{cell}$ remains constant. An increased concentration of H_2_O_2_ leads to enhanced lactate production. This suggests that the observed high lactate production in cancer cells does not occur solely because of the cell needs, but may also be related to the increase in the concentration levels of H_2_O_2_. We plot in the inset of Figure 3 the ratio between lactate production and glucose consumption against ROS concentration levels. This figure demonstrates that the larger portion of glucose is consumed by the cell metabolism, however, the corresponding share consumed by the PPP increases with elevations in the ROS concentration levels.

Notice that the presented results are qualitatively independent of the form of β and γ and we can derive the obtained results for any β and γ as long as they increase with increasing H_2_O_2_. These functions could be measured *in vitro* by putting different cell lines in a steady state flow of H_2_O_2_ and measuring the production rate of GSH for different concentrations of GSH. We also note that, at the same concentration of H_2_O_2_, glycolytic cells produce less GSH than cells that use oxidative phosphorylation.

In Shi et al. (2009) showed that by enhancing H_2_O_2_ in hepatoma cells, glycolysis activity increases, and by reducing H_2_O_2_ levels, this activity decreases. These observations are consistent with our prediction of a concomitant increase in glycolysis activity with increase in H_2_O_2_ and vice versa. We also note that Brand and Hermfisse (1997) observe that for proliferating rat thymocytes, cells switch to glycolysis to protect themselves against H_2_O_2_.

**Figure 4:**
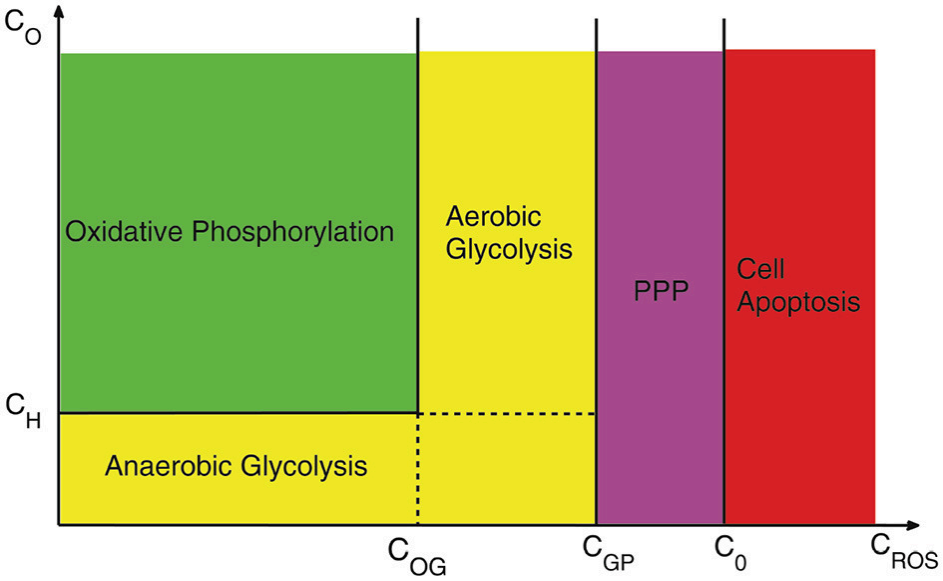
**A schematic of cell behavior under different concentrations of oxygen and H_**2**_O_**2**_**. *C*_*OG*_, *C*_*GP*_ are respectively the critical concentration for transition from oxidative phosphorylation to glycolysis and from Glycolysis to PPP and *C*_*H*_ is the transition point to hypoxia. The scales are for purely illustrative proposes and do not correspond to experimental values.

In Figure 4 we illustrate how the different dominant mechanisms in cell metabolism and detoxification evolve as the concentrations of oxygen and H_2_O_2_ vary. For concentrations of oxygen less than the hypoxic concentration (*C*_*H*_) and *C*_*ROS*_ less than the critical value for transition from oxidative phosphorylation to glycolysis (*C*_*OG*_), the metabolism is anaerobic glycolysis. When the concentration of oxygen passes the hypoxic concentration levels, cells transit to oxidative phosphorylation or aerobic glycolysis depending on whether *C*_*ROS*_ is less or greater than *C*_*OG*_. As the concentration of H_2_O_2_ increases and exceeds *C*_*GP*_, cells close all their metabolic pathways to drive the whole consumption of glucose toward PPP in order to reduce cell damage by H_2_O_2_. However, this process cannot continue indefinitely because changing glucose to G6P is ATP-dependent. Thus, cells need to keep their glycolytic metabolism active in order to continue the process of generating GSH and for detoxification of H_2_O_2_. When the concentrations of H_2_O_2_ exceed the critical concentration *C*_0_ tumor cells undergo apoptosis. Notice that this diagram is based on the most efficient mechanism of producing ATP and the availability of oxygen. It is possible that mutated cells activate less dominant metabolic pathways in any of these regions.

When some cells adopt the glycolytic phenotype, they have chosen the most efficient way of generating energy under H_2_O_2_ stress and are more resistant to ROS. Hence, at high concentrations of H_2_O_2,_ they have a higher survival advantage as compared to cells that rely on respiration. As a result, the glycolytic phenotypic population becomes the dominant population under ROS stress. Switching to the glycolytic phenotype may be realized through overexpression of glycolytic agents. In fact, experimental results report that PKM2 is activated in cancer cells which serves to shift the metabolism from oxidative phosphorylation to aerobic glycolysis (Christofk et al., 2008; Hitosugi et al., 2009). Interestingly, as the concentration of H_2_O_2_ gets close to the CSC (at which cell damage may occur), PKM2 is inhibited to drive the whole glucose flux to the PPP pathway and thus minimize the adverse effects of ROS (Anastasiou et al., 2011). Hence, PKM2 maybe be one of the primary candidates driving the described transition from oxidative phosphorylation to glycolysis.

We anticipate that there are other physiological and pathological situations in which our results might be pertinent and might help to explain certain biological behaviors. Two such examples are the observation of the glycolysis metabolism in embryos (Kondoh et al., 2007) and skeletal muscle (Richardson et al., 1998) which could well be described and understood based on our proposed model. Another example is the observation of the transition between glycolysis and oxidative phosphorylation in yeast (Chen et al., 2007). However, further investigations and more detailed discussion of these systems is beyond the scope of the current manuscript.

In conclusion, we have demonstrated that aerobic glycolysis is energetically more favorable than oxidative phosphorylation when the concentration levels of H_2_O_2_ exceed a certain critical value. This is because the energy generated by mitochondria is consumed by PPP to respond to the production and high concentrations of H_2_O_2_, generated by mitochondria. This makes oxidative phosphorylation an inefficient source of energy since it results in high energy consumption in order to respond to the production of H_2_O_2_ by mitochondria under high concentrations of H_2_O_2_. We have also shown that by increasing H_2_O_2_ levels, cells need to increase their glucose consumption via the glycolysis metabolism and PPP in order to satisfy their nutritional needs and for the purposes of removing H_2_O_2_. Thus, we propose that H_2_O_2_ is the major player behind the observed shift toward aerobic glycolysis in proliferating cancer cells.

## Author contributions

HM, MK and SS conceived and designed the research project. HM carried out the calculations and simulations. HM, MK and SS analyzed the results and wrote the manuscript.

## Funding

SS and MK acknowledge NSERC funding through individual Discovery Grants.

### Conflict of interest statement

The authors declare that the research was conducted in the absence of any commercial or financial relationships that could be construed as a potential conflict of interest.
