# Supplementary material for: High Concentrations of H2O2 Make Aerobic Glycolysis Energetically More Favorable for Cellular Respiration
Source: Front Physiol. 2016 Aug 23;7:362. doi: 10.3389/fphys.2016.00362 (PMC4993762; doi:10.3389/fphys.2016.00362)
Supplement: Supplementary file 1 [file DataSheet1.docx]

**Supplementary Information**

**Model**

We assume a minimal model, which includes the major pathways and species, in which the metabolism and detoxification pathways are oxidative phosphorylation, glycolysis and pentose phosphate pathway (PPP), which detoxifies H_2_O_2_. In this model we consider that cancer cells are in a homogeneous microenvironment and that cells consume oxygen and glucose at some constant rates and produce ATP and waste products. We also assume that growth factors and mitochondria produce H_2_O_2_ at some constant rate and that cells only use PPP to produce GSH to remove H_2_O_2_. In our model ATP, GSH and H_2_O_2_ are the main players, and although the effects of other anti-oxidative enzymes and ROS are important they do not affect the generality of our discussion.

We begin by deriving the kinetic equations for the chemical species that are involved in these processes. The three reactions that contribute to the process of generating GSH via PPP are:

$$Glucose +ATP \to G6P+ADP \left( 1 \right)$$

$$G6P+{2NADP}^{+}+H_{2}O \to R5P+2NADPH+2H^{+}+CO_{2} (2)$$

$GSSG+NADPH+H^{+}\to2GSH+{NADP}^{+} (3)$

The produced GSH participates in a chain of reactions that result in detoxification of H_2_O_2_ via the following reactions [1],

${GPx}_{r}+H_{2}O_{2}+H^{+}\underset{\to}{k_{1}}{GPx}_{0}+H_{2}O$ (4a)

${GPx}_{0}+GSH\underset{\to}{k_{2}}\left[ GS-GPx \right]+H_{2}O$ (4b)

$\left[ GS-GPx \right]+GSH\underset{\to}{k_{3}}{GPx}_{r}+GSSG+H^{+}$ (4c)

These reactions can be simplified to the following reaction,

$H_{2}O_{2}+2GSH\underset{\to}{GPx}GSSG+2H_{2}O (5)$

Also by integrating out the intermediate steps in equations (1-3) we derive the following reaction for the production of GSH,

$$Glucose+ATP+H_{2}O+2GSSG \to R5P+4GSH+CO_{2}+ADP (6)$$

In this equation $R5P$ may be used for synthesis of nucleotides and nucleic acids, which are necessary for cell proliferation, and GSH is used in Eq. (5) to detoxify H_2_O_2_. This reaction also needs one ATP to continue its process, hence it is coupled to the cell metabolism. Here, without loss of generality, we assume that glycolysis and oxidative phosphorylation are the only active metabolisms, therefore the chemical reactions representing the cell metabolisms read:

$Glucose+6O_{2}\to6CO_{2}+6H_{2}O (\text{energy}=36 ATP) Respiration (7)$

$$Glucose\to{2Lactate}^{-}+2H^{+} (\text{energy}=2 ATP) Glycolysis (8)$$

Assuming that the consumption rates of glucose, oxygen, and the production rates of lactate, ATP and H_2_O_2_ are respectively $q_{G}$, $q_{O},$ $P_{lact}$, $P_{ATP}$ and, $P_{ROS}$ and the concentration of glucose, ATP and H_2_O_2_ are respectively $C_{G}$, $C_{ATP}$ and $C_{ROS}$, in the following we derive the production rates of ATP, glucose and lactate.

As discussed in the manuscript, H_2_O_2_ can be produced by growth factors and mitochondria, hence

$$P_{ROS}= P_{ROS}^{mt}+P_{ROS}^{ext} (9)$$

Here $P_{ROS}^{mt}$ and $P_{ROS}^{ext}$are respectively the production rates of H_2_O_2_ by mitochondria and any other external sources such as growth factors. The production rate of H_2_O_2_ by mitochondria is a small fraction of the consumption rate of oxygen,

$$P_{ROS}^{mt}= \alpha q_{O} (10)$$

where $q_{O}$is the production rate of oxygen and $\alpha$ is the ratio of oxygen consumption to H_2_O_2_ production which is about 1/100-2/100 [3]. In response to this cells generate GSH at the following rate

$P_{GSH}=\beta P_{ROS}^{mt}+\gamma P_{ROS}^{ext}$ (11)

where $P_{GSH}$ is the production rates of GSH and $\beta$ and $\gamma$ are functions of the concentration of GSH and, the difference between CSC ($C_{0}$) and the concentration of H_2_O_2_ ($C_{ROS}$).

The net amount of ATP which is available to a cell is the sum of the ATP which is generated by oxidative phosphorylation and glycolysis minus the amount of ATP which is consumed through the detoxification of H_2_O_2_. In the following we calculate the net ATP produced by cell.

We start off by calculating the production rate of ATP by glycolysis. The amount of glucose which is consumed through glycolysis is the total consumption rate of glucose ($q_{G}$) minus the consumption rate of glucose by respiration ($\frac{q_{O}}{6}$) and detoxification ($\frac{P_{GSH}}{4}$), hence the production rate of ATP through glycolysis is:

$$P_{ATP}^{glycolysis}=2\left( q_{G}-\frac{q_{O}}{6}-\frac{P_{GSH}}{4} \right) (12)$$

The net ATP produced by a cell is the total of the ATP produced by respiration ($6q_{O}$) and glycolysis ($P_{ATP}^{glycolysis}$) minus the ATP consumed through detoxification ($\frac{P_{GSH}}{4}$), hence the total production rate of ATP reads,

$$P_{ATP}=6q_{O}+P_{ATP}^{glycolysis}-\frac{P_{GSH}}{4} (13)$$

Substituting Equation (11) into (12) and, Equations (11) and (12) into Equation (13) we obtain,

$$P_{ATP}=6q_{O}+2\left( q_{G}-\frac{q_{O}}{6}-\frac{\alpha\beta q_{O}+\gamma P_{ROS}^{ext}}{4} \right)-\frac{\alpha\beta q_{O}+\gamma P_{ROS}^{ext}}{4} (14)$$

This equation can be rewritten in the following form,

$$P_{ATP}=\left( \frac{17}{3}-\frac{3}{4}\alpha\beta\right)q_{O}+2q_{G}-\frac{3\gamma P_{ROS}^{ext}}{4} (15)$$

We define $r= \frac{q_{O}}{(q_{G}-q_{G}^{ROS})}$, where $q_{G}^{ROS}=\frac{\alpha\beta q_{O}+\gamma P_{ROS}^{ext}}{4}$ is the consumption rate of glucose through the detoxification channels and $r$ is a constant that can vary between 0 and 6, where zero corresponds to only glycolysis and six to purely respiration. Using these equations for $r$ and $q_{G}^{ROS}$, we can calculate $q_{O}$as function of $r$ as follows

$q_{O}=\frac{r(q_{G}-\frac{\gamma}{4}P_{ROS}^{ext})}{1+\frac{r\alpha\beta}{4}}$ (16)

Substituting this into Equation (15) gives,

$$P_{ATP}=\frac{\left( \frac{17}{3}-\frac{3}{4}\alpha\beta\right)(q_{G}-\frac{\gamma}{4}P_{ROS}^{ext})}{1+\frac{r\alpha\beta}{4}}r+2q_{G}-\frac{3\gamma P_{ROS}^{ext}}{4} (17)$$

Notice that the condition $q_{G}>\frac{3\gamma}{8}P_{ROS}^{ext}$ is always satisfied because the cell consumption of glucose through detoxification and its energy needs cannot exceed the total consumption of glucose. We now consider a scenario in which the consumption rate of ATP is equal to the cell requirements and derive the consumption of glucose as a function of the concentration and consumption rate of H_2_O_2_. Using this condition we obtain the following equation,

$q_{G}^{S}=\frac{(9+17r)\gamma}{24+68r-3r\alpha\beta}P_{ROS}^{ext}+\frac{12+3r\alpha\beta}{24+68r-3r\alpha\beta}q_{ATP}^{cell}$ (18)

where $q_{ATP}^{cell}$ and $q_{G}^{SS}$ are respectively the ATP and glucose consumption of the cell in steady state. Notice that in this equation $\beta$ is a function of $r$ because the production of H_2_O_2_ by mitochondria causes an increase in the total concentration of H_2_O_2_. We use Equation (18), and $P_{Lact}^{r=0}=2[q_{G}^{S}(r=0)-q_{G}^{ROS}]$, to derive the following equation for the total amount of lactate production in the case of pure glycolysis ($r=0$),

$$P_{Lact}^{r=0}=q_{ATP}^{cell}+{\frac{\gamma}{4}P}_{ROS}^{ext} (19)$$

References:

1. Ng, C. F., Schafer, F. Q., Buettner, G. R. & Rodgers, V. G. J. The rate of cellular hydrogen peroxide removal shows dependency on GSH: mathematical insight into in vivo H2O2 and GPx concentrations. *Free Radic Res.* **41,** 1201-11 (2007).

2. Li, S., Yan, T., Yang, J. Q., Oberley, T. D. & Oberley L. W. The Role of Cellular Glutathione Peroxidase Redox Regulation in the Suppression of Tumor Cell Growth by Manganese Superoxide Dismutase. *Cancer Research* **60,** 3927-3939 (2000).

3. Turrens, J. F. Mitochondrial formation of reactive oxygen species. *J. Physiol* **552,** 335-344 (2003).
